# Increased Overall Heart Rate Irregularity Risk by Hyperuricemia in the General Population: Results from the Korean National Health and Nutrition Examination Survey

**DOI:** 10.3390/medicina56100501

**Published:** 2020-09-24

**Authors:** Yeonghee Eun, Kyung-Do Han, Da Hye Kim, In Young Kim, Eun-Jung Park, Seulkee Lee, Hoon-Suk Cha, Eun-Mi Koh, Jaejoon Lee, Hyungjin Kim

**Affiliations:** 1 Department of Medicine, Samsung Medical Center, Sungkyunkwan University School of Medicine, Seoul 06351, Korea; eunbori03.work@gmail.com (Y.E.); seulkeelee.lee@samsung.com (S.L.); hoonsuk.cha@samsung.com (H.-S.C.); eunmi.koh@samsung.com (E.-M.K.); 2 Department of Statistics and Actuarial Science, Soongsil University, Seoul 06978, Korea; hkd@ssu.ac.kr (K.-D.H.); dahaene1228@naver.com (D.H.K.); 3 Department of Medicine, National Police Hospital, Seoul 05715, Korea; ariatansi@naver.com; 4 Department of Medicine, National Medical Center, Seoul 04564, Korea; ejcoke.park@gmail.com; 5 Department of Medical Humanities, Samsung Medical Center, Sungkyunkwan University School of Medicine, Seoul 06351, Korea

**Keywords:** hyperuricemia, arrhythmia, hypertension, heart rate, uric acid

## Abstract

*Background and objectives:* Hyperuricemia is one of the well-known cardiovascular risk factors. There is a growing interest in the association between hyperuricemia and arrhythmia. We used the representative sample data of Korean population to study the association between hyperuricemia and heart rate irregularity (HRI) that reflects total arrhythmia. *Materials and Methods:* We performed weighted multivariate logistic regression analysis to assess the association between hyperuricemia and HRI. *Results:* Of the 10,827 subjects, 1308 (13.2%) had hyperuricemia and 130 (1%) had HRI. In the presence of hyperuricemia, HRI was three times higher than that in the absence of hyperuricemia (OR 2.98, 95% CI 1.71–5.18). The risk of HRI was highest in subjects with both hypertension and hyperuricemia. In the subgroup analysis, the association of hyperuricemia with HRI was most pronounced in the smoker group. *Conclusions:* Hyperuricemia was highly correlated with HRI in adult Korean representative sample data. Hyperuricemia was associated with a nearly tripled risk for HRI. Hypertension has a synergistic effect with hyperuricemia on HRI. Further research is warranted to clarify the relationship between hyperuricemia and arrhythmia and its mechanism.

## 1. Introduction

Uric acid is an end product of purine metabolism in the human body. Uricase, an enzyme that degrades uric acid, is inactivated in humans, unlike in other mammalian species, leading to an increase in the concentration of serum uric acid (SUA) [1]. The effect of the increase in SUA levels in humans has been proposed to contribute to the potential antioxidant effect as an adaptive response to oxidative stress and to bl0ood pressure maintenance [2,3]. However, high levels of SUA can also lead to potentially harmful conditions.

Hyperuricemia is not only a risk factor of gout but has also recently been shown to be associated with cardiovascular disease and mortality [4,5,6,7,8]. In recent systematic reviews and meta-analysis, it was shown that hyperuricemia is associated with a modest elevation of hypertension risk [9,10] and risk of coronary heart disease events [7,11]. Meta-analysis of prospective cohort studies showed a 10% increase in coronary artery disease mortality and all-cause mortality per 1 mg/dl increase in SUA, and the increase in mortality was more pronounced in women [6,7].

Several studies have investigated the relationship between hyperuricemia and atrial fibrillation [12,13,14,15,16,17]. In a nationwide longitudinal cohort study conducted in Taiwan, hyperuricemia demonstrated a 1.2-fold increase in the risk of new-onset atrial fibrillation [12]. In a Tromso study, comprising a 11-year follow-up, prospective, and population-based cohort study of 6308 Norwegians, the hazard ratio of atrial fibrillation per 1 standard deviation (SD) increase in SUA (91 mmol/L) was 1.40 in women and 1.17 in men [14]. In addition to atrial fibrillation, a Japanese study of 167 patients with left ventricular hypertrophy detected by electrocardiogram demonstrated that the uric acid level was an independent predictive factor for ventricular arrhythmia occurrence [18]. Cicero et al. analyzed the data from the Brisighella Heart Study of 1639 patients and demonstrated that SUA is associated with sinus tachycardia and tachyarrhythmias [19].

Although there have been some studies on the association of hyperuricemia with sinus tachycardia or ventricular arrhythmia, data on the association between hyperuricemia and arrhythmias other than atrial fibrillation are still sparse. In this study, we used the representative sample data of adult Koreans to investigate the association between hyperuricemia and heart rate irregularity (HRI), which may reflect the overall arrhythmia.

## 2. Materials and Methods

### 2.1. Study Population

This cross-sectional study was based on the data from the seventh KNHANES, which was conducted by the Korean Centers for Disease Control and Prevention (KCDC) in 2016–2017. The KNHANES is designed to assess the health and nutritional status of the non-institutionalized civilian population in Korea. The survey includes health interviews, health examinations, and nutrition survey. KNHANES is conducted annually as a nationwide cross-sectional survey, and includes a new sample of approximately 10,000 individuals over the age of 1 year.

Of the 16,277 subjects who participated in KNHANES in 2016–2017, 3377 were excluded as they were under 19 years of age. We excluded 2073 participants who had missing data for the variables of interest. Finally, 10,827 subjects were included in the study.

### 2.2. Collection of Data

In the KNHANES, the data were obtained according to methods described in a previously published paper on data resource profile of KNHAES [20]. The health interview survey collected demographic and socioeconomic data, including age, sex, residential area, household income, and educational levels. Health-related habits, such as consumption of alcohol (frequency of alcohol use and drinking amount at one time), smoking (Smoking status, age at start of smoking, average amount of smoking per day, and days of smoking in the last month), and physical activity (physical exercise by intensity, number of workout days per week, and exercise time per day), and the prevalence of hypertension, diabetes, hyperlipidemia, chronic kidney disease (CKD), and cardiovascular disease (CVD) were also investigated. Hypertension was defined as taking antihypertensive medication, systolic blood pressure greater than 140 mmHg, or a diastolic blood pressure greater than 90 mmHg. Diabetes mellitus is defined as the use of hypoglycemic agents or insulin, fasting blood glucose level greater than 126 mg/dL, or a previous diagnosis by a physician. Hyperlipidemia is defined as taking lipid-lowering drugs or when fasting total cholesterol equal to or greater than 240 mg/dL. Chronic kidney disease is defined as an estimated glomerular filtration rate <60 mL/min/1.73 m^2^. Cardiovascular disease is defined as a diagnosis of stroke or myocardial infarction or angina by a physician.

Height, body weight, waist circumference, and blood pressure were measured and laboratory tests such as blood cell counts, fasting glucose, lipid profile, creatinine, uric acid, and high sensitivity C-reactive protein (hs-CRP) were performed when subjects participated in the survey. Subjects had blood tests after fasting after dinner the day before the investigation. According to standardized protocols, all health examination procedures were performed by trained medical personnel and all equipment was calibrated periodically. The body mass index was calculated as weight in kilograms divided by height in meters squared.

In order to measure the pulse rate in a stable state, the subject was allowed to sit in a chair for 5 min to rest and then pulse rate was measured. The trained examiner manually measured right radial pulse of the subject for 15 s. In the case of irregular pulse, bradycardia (<15 beats for 15 s), or tachycardia (>26 beats for 15 s), pulse rate was measured for 60 s to check regularity (Supplementary Information S1). Heart rate irregularity was defined as the variability of beat-by-beat intervals measured by the examiner for 60 s, including tachycardia and bradycardia, and cases where the beat-by-beat intervals were irregular within the normal heart rate range. The investigator was trained to determine the variability of beat-by-beat intervals by palpating radial pulses and to check yes or no on the recording sheet. Hyperuricemia was defined as a SUA level ≥7.0 mg/dL in men and ≥6.0 mg/dL in women.

### 2.3. Statistical Analysis

KNHANES was performed in a complex, stratified, multistage cluster sampling design, and design-based analyses were performed [21]. Data are presented as weighted means ± standard error (SE) for continuous variables or as weighted percentages (SE) for categorical variables. Only triglyceride values that did not satisfy the normality were expressed with geometric means and a 95% confidence interval. For variables that do not satisfy normality, logarithmic transformation was used. Student’s t-test, the chi-square test, and the Cochran–Armitage trend test were used for the comparison of demographic, socioeconomic, anthropometric, and laboratory variables according to the presence or absence of hyperuricemia and HRI. We performed weighted multivariate logistic regression analysis to assess the association between hyperuricemia and HRI. The dependent variable was HRI, and the independent variable was hyperuricemia. We analyzed continuous variables with odds ratios presented per 1 mg/dl greater uric acid level and categorical variables with odds ratios presented by the presence of hyperuricemia. The covariates used in the adjustment were age, sex, body mass index, smoking, drinking, hypertension, diabetes mellitus, and CVD. In order to determine whether age, sex, diabetes mellitus, chronic kidney disease, CVD, smoking and alcohol have a potential modification effect on the association between hyperuricemia and HRI, stratified analyses and interaction testing were performed using a likelihood ratio test by subgrouping. Subgroup analysis was adjusted for age, sex, body mass index, smoking, drinking, physical activity, and chronic kidney disease. Statistical significance was set at *p* < 0.05. All statistical analyses were conducted using SAS, version 9.2 (SAS Institute, Cary, NC, USA).

### 2.4. Ethics Statement

The KNHANES VII was carried out following approval from the institutional review board of the KCDC. All participants signed an informed consent form. The present study protocol conformed to the ethical guidelines of the 1975 Declaration of Helsinki, as revised in 1983, and was approved by the Institutional Review Board of Samsung Medical Center (number: SMC 2018-08-176).

## 3. Results

### 3.1. Characteristics of Study Population

Of the 10,827 participants, 1308 (13.2%) had hyperuricemia and 130 (1%) had HRI. The characteristics of the study population were analyzed according to the presence of hyperuricemia and HRI (Table 1, Tables S1 and S2). The hyperuricemic group had a higher proportion of males, smokers, and a higher prevalence of several comorbidities such as obesity, hypertension, hyperlipidemia, and chronic kidney disease than the non-hyperuricemic group. There was no significant difference in the prevalence of diabetes mellitus according to hyperuricemia, but differences were found when the non-diabetic category was subdivided into non-diabetic and impaired fasting glucose groups. This difference was mainly due to the high rate of impaired fasting glucose in subjects with hyperuricemia. The HRI was higher in the hyperuricemic group than in the non-hyperuricemic group (2.1% vs. 0.8%, *p* < 0.0001), but the prevalence of cardiovascular disease was similar in the two groups.

Subjects with irregular heart rate were older, more likely to be male, and had a higher proportion of former smokers than those with regular heart rate. The prevalence of concomitant diseases such as hypertension, diabetes, chronic kidney disease, and cardiovascular disease were higher in subjects with HRI than in those without HRI. The presence of hyperuricemia was also significantly different in the irregular heart rate group and regular heart rate group, respectively (29.5% vs. 13.1%, *p* < 0.0001).

### 3.2. The Association of Hyperuricemia and HRI

To investigate whether hyperuricemia and HRI are related, we performed multivariate regression analysis (Table 2). The risk of HRI was observed to increase 1.3 times for every 1 mg/dL increase in serum uric acid level when analyzing uric acid as a continuous variable. Using the categorical variable, namely the presence of hyperuricemia, the odds of HRI were observed to increase by nearly three times with hyperuricemia. This association remained significant even when adjusted for confounding factors such as age, sex, body mass index, smoking, drinking, hypertension, diabetes, and cardiovascular disease. The association was significant even when the adjustment was modified by adding other variables such as chronic kidney disease and physical activity and changing the combinations of factors. When multivariate regression analyses were performed separately for men and women, the association between HRI and hyperuricemia was confirmed regardless of gender (Tables S3 and S4).

To determine the effect of too low or too high uric acid level on HRI, the odds ratio of HRI according to uric acid level is shown in Table S5. The association between uric acid and HRI was most pronounced when uric acid level was above 9 mg/d (*p* for trend = 0.0019).

### 3.3. Effects of Hypertension and Hyperuricemia on HRI

**Figure 1:**
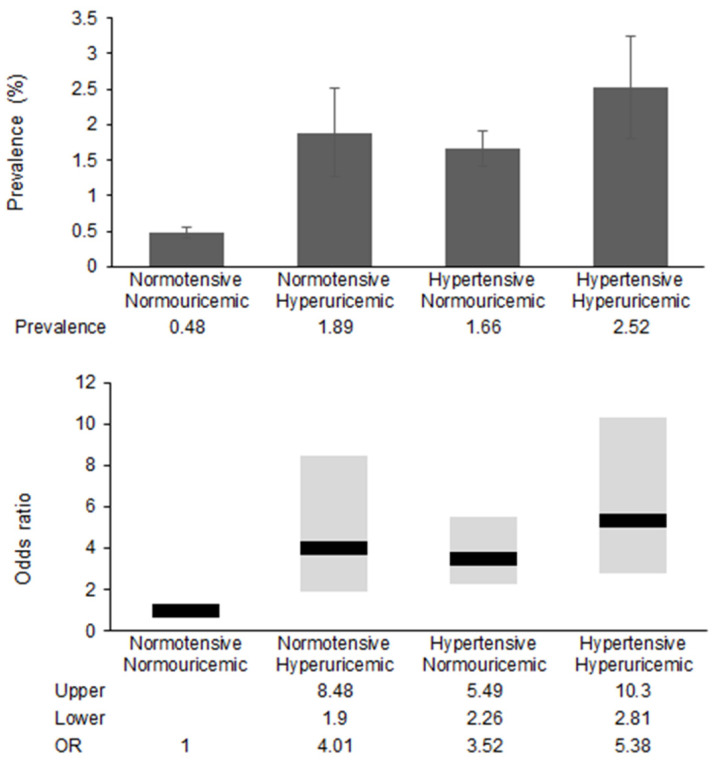
Differences in the prevalence and odds ratio of heart rate irregularity.

Figure 1 shows the prevalence and odds ratios of HRI according to the presence of hypertension and hyperuricemia. In normotensive subjects, hyperuricemia increased the odds ratio of HRI by 4-fold, and in normouricemic subjects, hypertension increased the odds ratio of HRI by 3.5-fold. The risk of HRI is five times higher in subjects with both hypertension and hyperuricemia than in those without both.

### 3.4. Subgroup Analyses According to Baseline Characteristics

**Figure 2:**
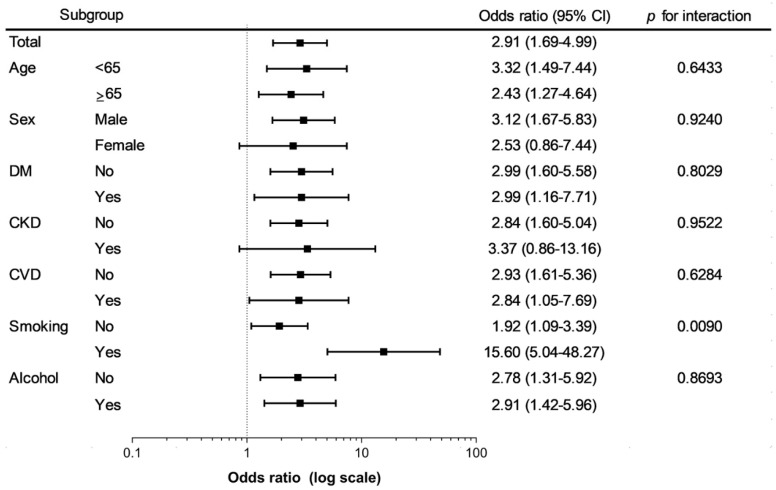
Odds ratio and 95% confidence interval for the heart rate irregularity in subjects with hyperuricemia compared to those without hyperuricemia. Adjusted for age, sex, body mass index, smoking, drinking, physical activity and CKD. Abbreviations: DM, diabetes mellitus; CKD, chronic kidney disease; CVD, cardiovascular disease; 95% CI, 95% confidence interval.

In subgroup analyses, there was a significant heterogeneity in the odds ratios for HRI associated with hyperuricemia in smokers and non-smokers (Figure 2). Hyperuricemia was associated with significantly higher odds of HRI in smokers when compared to non-smokers (*p* for interaction = 0.0090). In other subgroup analyses, there was no difference in the association between hyperuricemia and HRI.

## 4. Discussion

This study demonstrates the association between hyperuricemia and HRI using nationally representative data of South Korea. In this study, the subjects with hyperuricemia showed an almost 3-fold increase in risk of HRI compared to those without hyperuricemia. This association was statistically significant after adjustments for age, sex, body mass index, smoking, drinking, and comorbidities.

A considerable number of epidemiologic studies have reported an association between hyperuricemia and atrial fibrillation. In a 5.4-year follow-up health screening program cohort study of approximately 280,000 healthy people in South Korea, an increase in the SUA level was significantly and positively associated with incident atrial fibrillation [22]. Meta-analyses showed that hyperuricemia was associated with an increased risk of atrial fibrillation [23,24,25]. Although the number of studies is less than that of atrial fibrillation, studies on other types of arrhythmias have been reported. Furthermore, elevated uric acid levels were associated with a nearly 2-fold increase in the risk of cardiac conduction defects in hospitalized patients with type 2 diabetes [26]. Yamada et al. showed that the uric acid level was an independent predictive factor for the occurrence of ventricular tachycardia in patients with left ventricular hypertrophy detected by electrocardiography [18]. The results of this study are also in line with those of previous studies, demonstrating that hyperuricemia shows a positive association with HRI, an indicator of overall arrhythmia.

The mechanism by which uric acid promotes arrhythmia is still yet to be elucidated. Several plausible mechanisms have been proposed for the contribution of hyperuricemia to arrhythmia [27,28,29]. Monosodium urate crystals, which are formed by the precipitation of high concentrations of uric acid, act as a danger signal and activate the Nod-like receptor family protein 3 (NLRP3) inflammasome pathway. This pathway is involved in the secretion of interleukin (IL)-1β from human macrophages [30]. IL-1β promotes myofibroblast differentiation and collagen deposition leading to fibrosis [31,32,33]. The NLRP3 inflammasome is upregulated in the atrial tissue of atrial fibrillation patients and the inhibition of NLRP3 reduces atrial fibrillation inducibility in cardiomyocyte-specific knock-in mouse models expressing constitutively active NLRP3 [34]. In addition, intracellular uric acid taken up by uric acid transporters increases the oxidative stress in atrial myocytes, which can activate the Ca^2+^-permeable transient receptor potential melastatin-related type 7 channels (TRPM7s) [35]. TRMP7s are upregulated in the atrial fibroblasts of atrial fibrillation patients and contribute to the transforming growth factor (TGF)-β1-induced fibroblast differentiation. In cultured mouse atrial myocytes, the intracellular urate promotes the expression of K+ channels, which may increase the risk of tachyarrhythmias [36].

Hypertension is a common cardiovascular risk factor, and hypertensive heart disease can manifest as several cardiac arrhythmias [37,38]. Hypertension can lead to left ventricular hypertrophy and myocardial fibrosis and can trigger arrhythmia by activating the sympathetic nervous system and the renin–angiotensin–aldosterone system [39,40]. Mechanical overload due to high blood pressure may induce an abnormal expression of ion channels and/or junctional complexes [41]. In our study, subjects with both hypertension and hyperuricemia had higher HRI prevalence and odds ratios than those without both. Since both hypertension and hyperuricemia can induce myocardial fibrosis and ion channel changes, hypertension and hyperuricemia could have a synergistic effect on HRI. Further research is needed to clarify the mechanism by which hypertension and hyperuricemia cause synergistic effects.

In the subgroup analysis, most patient characteristics did not affect the association between HRI and hyperuricemia, with the exception in which smokers had a greater association between hyperuricemia and HRI. While the effects of smoking on the progression of atherosclerotic disease have been well studied, the role of smoking in cardiac arrhythmia is less clearly defined [42]. However, it is suggested that the pro-fibrotic effect of nicotine or effect on ion channels, and increased oxidative stress due to smoking may promote cardiac arrhythmias [43,44,45]. Hyperuricemia in smokers may promote atrial fibrosis and increase the oxidative stress, making them more prone to develop arrhythmias.

Our study has several limitations. First, this study was performed in a cross-sectional manner. It was possible to establish the association between hyperuricemia and HRI, but no causal relationship could be confirmed. Second, accurate characterization of arrhythmias was impossible due to the absence of electrocardiography information. Since the only information available in relation to arrhythmia in KNHANES was HRI, there is a lack of confidence in how much HRI can represent arrhythmia. However, since it is a measure consistently measured by experienced inspectors, it was assumed that it would reflect arrhythmia. The KNHANES is conducted by trained medical personnel according to standardized protocol, and the quality control is thoroughly conducted by experts and the government. Despite the limitation of some inaccuracies, HRI is an easily measurable indicator that can be easily applied in a real clinical setting. Third, KNHANES includes a questionnaire about the presence of hypertension and use of antihypertensive medications, but does not include specific information about antihypertensive medications. Therefore, it was impossible to adjust medications such as beta-blockers or calcium channel blockers that could affect the heart rate. However, when hypertension was adjusted in the regression model, the association between hyperuricemia and HRI was significant, and the association was maintained even with the adjustment of the use of antihypertensive medication. Fourth, this study was conducted only for Koreans, and there are limitations in applying the results to other races. Despite these limitations, this study would be meaningful in that we clearly demonstrate the association between hyperuricemia and arrhythmia using the large nation-wide representative sample data.

## 5. Conclusions

In conclusion, hyperuricemia was highly correlated with HRI in the adult Korean representative sample data. Hyperuricemia was associated with a nearly tripled risk for HRI. Hypertension has a synergistic effect with hyperuricemia on HRI. Further studies are required to elucidate the relationship between hyperuricemia and arrhythmia and its mechanism.

## Figures and Tables

**Table 1:** Characteristics of the study population.

	Total (*n =* 10,827)	Non-Hyperuricemic (*n =* 9519)	Hyperuricemic (*n =* 1308)	*p*	Regular HR (*n =* 10,697)	Irregular HR (*n =* 130)	*p*
Age, years	46.6 ± 0.3	47.0 ± 0.3	44.4 ± 0.6	<0.0001	46.5 ± 0.3	62.2 ± 1.9	<0.0001
Sex, male	50.0 (0.5)	45.9 (0.5)	76.6 (1.3)	<0.0001	49.9 (0.5)	62.8 (5.2)	0.0072
Urban residence	85.1 (1.7)	85.0 (1.7)	85.9 (1.9)	0.5320	85.1 (1.7)	88.4 (2.9)	0.2678
Occupation	64.8 (0.7)	64.4 (0.7)	67.4 (1.7)	0.0789	64.9 (0.7)	52.0 (5.4)	0.0139
Income (lowest quartile)	15.1 (0.7)	15.1 (0.7)	15.2 (1.3)	0.9276	15.1 (0.7)	24.2 (4.2)	0.0090
Low education level ^a^	23.0 (0.8)	23.5 (0.8)	19.6 (1.3)	0.0011	22.8 (0.8)	44.6 (4.9)	<0.0001
Smoking				<0.0001			<0.0001
Never	59.4 (0.6)	61.8 (0.6)	43.7 (1.6)		59.6 (0.6)	43.4 (5.2)	
Former	19.0 (0.4)	18.2 (0.4)	24.3 (1.4)		18.8 (0.4)	41.2 (5.1)	
Current	21.6 (0.6)	20.0 (0.6)	32.0 (1.5)		21.6 (0.6)	15.4 (4.6)	
Alcohol consumption				<0.0001			0.1677
None	22.1 (0.6)	23.1 (0.6)	15.9 (1.1)		22.1 (0.6)	29.9 (4.9)	
<30 g/day	68.7 (0.6)	68.8 (0.6)	67.8 (1.5)		68.7 (0.6)	63.5 (5.3)	
≥30 g/day	9.2 (0.3)	8.1 (0.3)	16.4 (1.3)		9.3 (0.3)	6.6 (2.5)	
Physical activity ^b^	47.6 (0.7)	47.0 (0.7)	51.7 (1.7)	0.0062	47.6 (0.7)	45.1 (5.6)	0.6466
BMI, kg/m^2^	23.9 ± 0.1	23.6 ± 0.1	25.9 ± 0.1	<0.0001	24.0 ± 0.1	23.7 ± 0.3	0.4654
WC, cm	82.2 ± 0.2	81.3 ± 0.2	88.3 ± 0.3	<0.0001	82.2 ± 0.2	84.1 ± 0.9	0.0301
HR, per minute	57.6 ± 0.6	57.2 ± 0.6	60.0 ± 2.1	0.1980	55.9 ± 0.6	68.5 ± 1.5	<0.0001
HR irregularity	1.0 (0.1)	0.8 (0.1)	2.1 (0.5)	<0.0001			
Systolic BP, mmHg	117.5 ± 0.2	116.9 ± 0.2	121.8 ± 0.5	<0.0001	117.5 ± 0.2	122.1 ± 1.6	0.0043
Diastolic BP, mmHg	75.9 ± 0.2	75.4 ± 0.2	79.4 ± 0.4	<0.0001	76.0 ± 0.2	73.0 ± 1.0	0.0034
Fasting glucose, mg/dL	99.5 ± 0.3	99.4 ± 0.3	100.5 ± 0.6	0.0720	99.5 ± 0.3	107.2 ± 3.3	0.0198
Total C, mg/dL	193.5 ± 0.5	192.3 ± 0.5	201.3 ± 1.4	<0.0001	193.6 ± 0.5	180.5 ± 3.6	0.0003
TG ^c^, mg/dL	112.4 (110.7–114.1)	106.9 (105.3–108.5)	155.8 (148.6–163.3)	<0.0001	112.4 (110.7–114.1)	109.8 (98.3–122.6)	0.6756
eGFR ^d^, mL/min/1.73m^2^	96.5 ± 0.3	97.7 ± 0.3	88.3 ± 0.6	<0.0001	96.6 ± 0.3	83.1 ± 2.4	<0.0001
Uric acid, mg/dL	5.14 ± 0.02	4.77 ± 0.01	7.53 ± 0.03	<0.0001	5.13 ± 0.02	5.66 ± 0.19	0.0072
hs-CRP, mg/dL	1.18 ± 0.02	1.12 ± 0.02	1.55 ± 0.07	<0.0001	1.17 ± 0.02	1.71 ± 0.34	0.1139
Obesity ^e^	34.9 (0.6)	31.6 (0.7)	56.4 (1.6)	<0.0001	34.9 (0.6)	35.9 (4.6)	0.8276
Abdominal obesity ^f^	28.3 (0.7)	25.5 (0.7)	47.0 (1.6)	<0.0001	28.3 (0.7)	32.7 (4.8)	0.3307
Hyperuricemia	13.2 (0.4)				13.1 (0.4)	29.5 (5.3)	<0.0001
Hypertension	26.9 (0.6)	25.1 (0.6)	38.4 (1.7)	<0.0001	26.7 (0.6)	51.4 (5.1)	<0.0001
Diabetes mellitus	10.4 (0.4)	10.3 (0.4)	10.6 (1.0)	0.7933	10.2 (0.4)	22.1 (3.9)	<0.0001
Hyperlipidemia	20.0 (0.5)	19.5 (0.5)	23.6 (1.4)	0.0032	20.0 (0.5)	18.3 (3.7)	0.6596
Chronic kidney disease	2.2 (0.2)	1.3 (0.1)	7.9 (0.7)	<0.0001	2.1 (0.2)	11.2 (3.1)	<0.0001
Cardiovascular disease	3.5 (0.2)	3.6 (0.2)	3.1 (0.5)	0.3184	3.4 (0.2)	15.9 (3.0)	<0.0001

Data are presented as weighted mean ± standard error (SE) or weighted percentage (SE). ^a^ Low education level means middle school graduate or less. ^b^ Physical activity refers to more than 150 min of moderate-intensity aerobic physical activity throughout the week, or more than 75 min of vigorous-intensity aerobic physical activity throughout the week, or an equivalent combination of moderate- and vigorous-intensity activity. ^c^ TG values are presented as a geometric mean (95% confidence interval). ^d^ An eGFR calculated from serum creatinine using an isotope dilution mass spectrometry (IDMS) traceable Modification of Diet in Renal Disease (MDRD) Study equation. ^e^ Obesity is defined as a BMI ≥25 kg/m^2^. ^f^ Abdominal obesity is defined as a WC >90 cm in men or >85 cm in women. Abbreviations: HR, heart rate; BMI, body mass index; WC, waist circumference; BP, blood pressure; C, cholesterol; TG, triglyceride; eGFR, estimated glomerular filtration rate; hs-CRP, high-sensitivity C-reactive protein.

**Table 2:** Multivariate regression analyses for association between hyperuricemia and heart rate irregularity.

	Prevalence % (SE)	OR (95% CI)			
		Model 1	Model 2	Model 3	Model 4
Uric acid, continuous (per mg/dL)		1.28 (1.09–1.51)	1.28 (1.07–1.53)	1.30 (1.08–1.57)	1.30 (1.08–1.57)
*p*		0.0029	0.0071	0.0049	0.0051
Hyperuricemia					
No	0.78 (0.09)	1.00 (ref.)	1.00 (ref.)	1.00 (ref.)	1.00 (ref.)
Yes	2.13 (0.47)	2.79 (1.70–4.57)	2.79 (1.68–4.63)	2.98 (1.71–5.18)	3.00 (1.72–5.24)
*p*	<0.0001	<0.0001	<0.0001	0.0001	0.0001

Model 1 is a non-adjusted model. Model 2 is adjusted for age and sex. Model 3 is adjusted for model 2 + body mass index, smoking, drinking, hypertension, and diabetes mellitus. Model 4 is adjusted for model 3 + cardiovascular disease. Cardiovascular disease is defined as a diagnosis of stroke or myocardial infarction or angina by a physician.

## Supplementary Materials

The following are available online at https://www.mdpi.com/1010-660X/56/10/501/s1, Information S1: Measurement of heart rate in the Korean National Health and Nutrition Examination Survey, Table S1: Characteristics of male participants in the study, Table S2: Characteristics of female participants in the study, Table S3: Multivariate regression analyses for association between hyperuricemia and heart rate irregularity in male population, Table S4: Multivariate regression analyses for association between hyperuricemia and heart rate irregularity in female population, Table S5: Odds ratio for heart rate irregularity according to uric acid level.
