# Supplementary material for: Increased Overall Heart Rate Irregularity Risk by Hyperuricemia in the General Population: Results from the Korean National Health and Nutrition Examination Survey
Source: Medicina (Kaunas). 2020 Sep 24;56(10):501. doi: 10.3390/medicina56100501 (PMC7600340; doi:10.3390/medicina56100501)
Supplement: Supplementary file 1 [file medicina-56-00501-s001.pdf]

medicina

Information S1. Measurement of heart rate in the Korean National Health and Nutrition Examination Survey.

1) Eligible and excluded subjects

Pulse of all subjects aged 10 years or older are measured. However, subjects with rashes, open wounds, weakness, splints, edema, hematomas, and arteriovenous fistula for dialysis are excluded from the measurement. Female subjects underwent axillary node biopsy or surgery, or one radical mastectomy, measured on the opposite arm. Female subjects who underwent both radical mastectomies are excluded from the measurement.

2) Preparation before pulse measurement

A. Sitting posture

Sit back against the back of the chair so that the subject's spine is in a straight line and help them sit stably and comfortably. Do not cross the legs and keep their feet flat on the floor. If their feet do not touch the floor, use a footrest.

B. Measuring arm selection

In principle, right arm measurement is used for standardization. However, if measurement is not possible on the right arm, the measurement is made on the left arm.

C. Precautions before measurement

To measure the pulse in a stable state, the subject must have a 5-minute rest period while sitting in a chair before measurement. Words, conversations, and movements can affect the measurement, so avoid them until the measurement is finished. After the 5-minute rest period, the pulse rate is measured.

3) Measurement of pulse rate

With the sleeve of the measuring arm fully rolled up, palms face up and the elbows are supported with armrests to keep slightly bent.

Measure and record the right radial pulse for 15 seconds. If the pulse is irregular or bradycardia (<15 beats for 15 seconds) or tachycardia (>26 beats for 15 seconds), check the pulse rate for 60 seconds and check whether it is regular and record it.

Table S1. Characteristics of male participants in the study.

	Total (n=4756)	Non-hyperuricemic (n=3847)	Hyperuricemic (n=909)	*p*	Regular HR (n = 4672)	Irregular HR (n = 84)	*p*
Age, years	45.7 ± 0.3	46.7 ± 0.4	41.8 ± 0.6	<0.0001	45.5 ± 0.3	62.5 ± 2.3	<0.0001
Urban residence	85.1 (1.7)	84.5 (1.8)	87.2 (1.9)	0.0947	85.0 (1.7)	88.3 (3.7)	0.4077
Occupation	76.2 (0.8)	76.1 (0.9)	76.4 (1.8)	0.8907	76.4 (0.9)	60.3 (6.3)	0.0042
Income (lowest quartile)	13.7 (0.8)	14.1 (0.8)	12.0 (1.4)	0.1598	13.5 (0.8)	22.2 (4.6)	0.0238
Low education level[a]	17.8 (0.8)	18.8 (0.9)	14.2 (1.2)	0.0014	17.5 (0.8)	46.6 (6.2)	<0.0001
Smoking				0.1222			<0.0001
Never	28.9 (0.8)	28.4 (0.9)	30.9 (1.9)		29.0 (0.9)	17.0 (4.2)	
Former	33.4 (0.8)	34.2 (0.9)	30.3 (1.8)		33.1 (0.8)	61.0 (6.7)	
Current	37.8 (1.0)	37.5 (1.0)	38.9 (1.9)		37.9 (1.0)	22.1 (6.2)	
Alcohol consumption				0.0003			0.1317
None	13.8 (0.6)	14.6 (0.7)	10.8 (1.1)		13.7 (0.6)	21.4 (4.8)	
<30 g/day	70.4 (0.8)	70.7 (0.9)	69.1 (1.7)		70.4 (0.8)	68.1 (6.1)	
≥30 g/day	15.8 (0.6)	14.8 (0.7)	20.0 (1.6)		15.9 (0.6)	10.5 (3.8)	
Physical activity[b]	50.7 (1.0)	50.0 (1.1)	53.7 (2.0)	0.0883	50.7 (1.0)	51.8 (6.6)	0.8786
BMI, kg/m^2	24.6 ± 0.1	24.2 ± 0.1	25.9 ± 0.1	<0.0001	24.6 ± 0.1	23.9 ± 0.4	0.0721
WC, cm	86.1 ± 0.2	85.3 ± 0.2	89.1 ± 0.4	<0.0001	86.1 ± 0.2	85.8 ± 1.1	0.8080
HR, per minute	57.1 ± 0.7	56.3 ± 0.7	60.6 ± 2.5	0.0968	55.4 ± 0.8	68.4 ± 1.9	<0.0001
HR irregularity	1.2 (0.2)	1.0 (0.1)	2.0 (0.5)	0.0140			
Systolic BP, mmHg	120.0 ± 0.3	119.5 ± 0.3	122.2 ± 0.5	<0.0001	120.0 ± 0.3	121.1 ± 2.2	0.5919
Diastolic BP, mmHg	78.5 ± 0.2	77.9 ± 0.2	80.8 ± 0.4	<0.0001	78.6 ± 0.2	72.4 ± 1.2	<0.0001
Fasting glucose, mg/dL	101.8 ± 0.5	102.4 ± 0.5	122.2 ± 0.5	<0.0001	101.8 ± 0.5	104.5 ± 2.3	0.2447
Total C, mg/dL	192.4 ± 0.7	190.2 ± 0.7	201.0 ± 1.7	<0.0001	192.6 ± 0.7	175.9 ± 4.7	0.0004
TG[c], mg/dL	131.7 (128.8-134.6)	124.4 (121.7-127.2)	164.7 (155.8-174.1)	<0.0001	132.0 (129.1-134.9)	111.2 (96.7-127.9)	0.0172
eGFR[d], mL/min/1.73m^2	93.9 ± 0.4	95.0 ± 0.4	89.7 ± 0.6	<0.0001	94.1 ± 0.4	83.5 ± 2.4	<0.0001
Uric acid, mg/dL	5.90 ± 0.03	5.42 ± 0.02	7.81 ± 0.03	<0.0001	5.90 ± 0.03	6.25 ± 0.23	0.1229
hs-CRP, mg/dL	1.29 ± 0.03	1.24 ± 0.04	1.47 ± 0.07	0.0058	1.28 ± 0.03	2.04 ± 0.52	0.1476
Hyperuricemia[e]	20.2 (0.7)				20.1 (0.7)	34.0 (6.7)	0.0140

Hypertension[f]	30.9 (0.8)	29.5 (0.9)	36.4 (1.9)	0.0007	30.6 (0.8)	54.2 (6.7)	0.0002
Diabetes mellitus[g]	11.4 (0.5)	12.4 (0.6)	7.8 (0.9)	0.0002	11.3 (0.5)	21.9 (4.9)	0.0054
Hyperlipidemia[h]	8.4 (0.5)	8.9 (0.5)	6.2 (0.9)	0.0181	8.4 (0.5)	9.3 (3.1)	0.7596

Data are presented as weighted mean ± standard error (SE) or weighted percentage (SE). [a] Low education level means middle school graduate or less. [b] Physical activity is defined in accordance with the WHO recommendations, which refers to more than 150 minutes of moderate-intensity aerobic physical activity throughout the week, or more than 75 minutes of vigorous-intensity aerobic physical activity throughout the week, or an equivalent combination of moderate- and vigorous-intensity activity. [c] TG values are presented as a geometric mean (95% confidence interval). [d] An eGFR calculated from serum creatinine using an isotope dilution mass spectrometry (IDMS) traceable Modification of Diet in Renal Disease (MDRD) Study equation. [e] Hyperuricemia is defined as a serum uric acid level of ≥7.0 mg/dL for men. [f] Hypertension is defined as a systolic BP >140 mmHg, or a diastolic BP >90 mmHg, or taking antihypertensive medication. [g] Diabetes mellitus is defined as a fasting blood glucose level >126 mg/dL, or previous history of diagnosis by a doctor, or taking hypoglycemic agent or insulin. [h] Hyperlipidemia is defined as a fasting total C ≥240 mg/dL or taking a lipid lowering agent. Abbreviations: HR, heart rate; BMI, body mass index; WC, waist circumference; BP, blood pressure; C, cholesterol; TG, triglyceride; eGFR, estimated glomerular filtration rate; hs-CRP, high-sensitivity C-reactive protein.

Table S2. Characteristics of female participants in the study.

	Total (n=6071)	Non-hyperuricemic (n=5672)	Hyperuricemic (n=399)	*p*	Regular HR (n=6025)	Irregular HR (n=46)	*p*
Age, years	47.6 ± 0.3	47.2 ± 0.3	52.8 ± 1.2	<0.0001	47.5 ± 0.3	61.7 ± 3.1	<0.0001
Urban residence	85.2 (1.7)	85.4 (1.7)	81.4 (2.9)	0.0497	85.2 (1.7)	88.7 (4.5)	0.4783
Occupation	53.4 (0.9)	54.4 (0.9)	38.0 (2.8)	<0.0001	53.5 (0.9)	37.9 (8.6)	0.0791
Income (lowest quartile)	16.6 (0.8)	16.0 (0.8)	25.8 (2.5)	<0.0001	16.5 (0.8)	27.6 (7.0)	0.0582
Low education level[a]	28.2 (0.9)	27.6 (0.9)	37.3 (3.0)	0.0003	28.1 (0.9)	41.3 (7.2)	0.0422
Smoking				0.0038			0.6395
Never	89.9 (0.5)	90.2 (0.5)	85.6 (2.0)		90.0 (0.5)	88.2 (5.3)	
Former	4.7 (0.3)	4.7 (0.4)	4.8 (1.2)		4.7 (0.3)	7.7 (4.4)	
Current	5.4 (0.4)	5.1 (0.4)	9.6 (1.8)		5.4 (0.4)	4.1 (3.1)	
Alcohol consumption				0.1252			N/A
None	30.5 (0.8)	30.3 (0.8)	32.4 (2.7)		30.4 (0.7)	44.2 (8.9)	
<30 g/day	66.9 (0.8)	67.2 (0.8(	63.3 (2.8)		67.0 (0.8)	55.9 (8.9)	
≥30 g/day	2.6 (0.3)	2.5 (0.3)	4.3 (1.3)		2.7 (0.3)	N/A	
Physical activity[b]	44.5 (0.9)	44.4 (0.9)	45.2 (2.9)	0.7936	44.6 (0.9)	33.8 (9.3)	0.2702
BMI, kg/m^2	23.3 ± 0.1	23.1 ± 0.1	26.0 ± 0.3	<0.0001	23.3 ± 0.1	23.5 ± 0.6	0.7544
WC, cm	78.4 ± 0.2	77.9 ± 0.2	85.9 ± 0.7	<0.0001	78.4 ± 0.2	81.3 ± 1.5	0.0480
HR, per minute	58.5 ± 0.9	58.6 ± 1.0	57.3 ± 3.1	0.6941	56.8 ± 0.9	68.7 ± 2.1	<0.0001
HR irregularity	0.7 (0.1)	0.6 (0.1)	2.5 (1.1)	0.0011			
Systolic BP, mmHg	115.0 ± 0.3	114.6 ± 0.3	120.3 ± 1.1	<0.0001	114.9 ± 0.3	123.7 ± 2.3	0.0002
Diastolic BP, mmHg	73.4 ± 0.2	73.3 ± 0.2	75.1 ± 0.6	0.0060	73.4 ± 0.2	73.9 ± 1.8	<0.7677
Fasting glucose, mg/dL	97.2 ± 0.4	96.8 ± 0.4	103.2 ± 1.4	<0.0001	97.1 ± 0.4	111.8 ± 7.5	0.0501
Total C, mg/dL	194.6 ± 0.6	194.1 ± 0.6	202.3 ± 2.2	0.0002	194.6 ± 0.6	188.3 ± 6.0	0.2935
TG[c], mg/dL	95.9 (94.1-97.7)	94.0 (92.2-95.8)	129.7 (121.2-138.9)	<0.0001	95.8 (94.0-97.7)	107.4 (91.8-125.7)	0.1559
eGFR[d], mL/min/1.73m^2	99.0 ± 0.4	100.0 ± 0.4	83.8 ± 1.6	<0.0001	99.2 ± 0.4	82.6 ± 4.9	0.0007
Uric acid, mg/dL	4.37 ± 0.02	4.22 ± 0.01	6.65 ± 0.04	<0.0001	4.37 ± 0.02	4.66 ± 0.26	0.2595
hs-CRP, mg/dL	1.07 ± 0.02	1.02 ± 0.02	1.80 ± 0.13	<0.0001	1.06 ± 0.02	1.17 ± 0.22	0.6202
Hyperuricemia[e]	6.2 (0.4)				6.1 (0.4)	21.9 (8.5)	0.0011
Hypertension[f]	22.9 (0.8)	21.4 (0.7)	44.9 (3.1)	<0.0001	22.7 (0.8)	46.6 (8.6)	0.0011

Diabetes mellitus[g]	9.3 (0.5)	8.6 (0.5)	19.6 (2.4)	<0.0001	9.2 (0.5)	22.5 (6.8)	0.0048
Hyperlipidemia[h]	10.8 (0.5)	10.6 (0.5)	14.5 (2.0)	0.0299	10.7 (0.5)	25.6 (7.3)	0.0043

Data are presented as weighted mean ± standard error (SE) or weighted percentage (SE). [a] Low education level means middle school graduate or less. [b] Physical activity is defined in accordance with the WHO recommendations, which refers to more than 150 minutes of moderate-intensity aerobic physical activity throughout the week, or more than 75 minutes of vigorous-intensity aerobic physical activity throughout the week, or an equivalent combination of moderate- and vigorous-intensity activity. [c] TG values are presented as a geometric mean (95% confidence interval). [d] An eGFR calculated from serum creatinine using an isotope dilution mass spectrometry (IDMS) traceable Modification of Diet in Renal Disease (MDRD) Study equation. [e] Hyperuricemia is defined as a serum uric acid level of ≥7.0 mg/dL for men. [f] Hypertension is defined as a systolic BP >140 mmHg, or a diastolic BP >90 mmHg, or taking antihypertensive medication. [g] Diabetes mellitus is defined as a fasting blood glucose level >126 mg/dL, or previous history of diagnosis by a doctor, or taking hypoglycemic agent or insulin. [h] Hyperlipidemia is defined as a fasting total C ≥240 mg/dL or taking a lipid lowering agent. Abbreviations: HR, heart rate; BMI, body mass index; WC, waist circumference; BP, blood pressure; C, cholesterol; TG, triglyceride; eGFR, estimated glomerular filtration rate; hs-CRP, high-sensitivity C-reactive protein; N/A, not applicable.

medicina

Table S3. Multivariate regression analyses for association between hyperuricemia and heart rate irregularity in male population.

	Prevalence % (SE)	OR (95% CI)			
		Model 1	Model 2	Model 3	Model 4
Uric acid, continuous (per mg/dL)		1.22 (0.96-1.54)	1.34 (1.11-1.62)	1.35 (1.10-1.65)	1.38 (1.11-1.72)
p		0.1029	0.0028	0.0037	0.0038
Hyperuricemia					
No	0.99 (0.14)	1.00 (ref.)	1.00 (ref.)	1.00 (ref.)	1.00 (ref.)
Yes	2.02 (0.51)	2.05 (1.14-3.69)	2.87 (1.61-5.12)	2.91 (1.55-5.46)	3.04 (1.61-5.74)
p	0.0140	0.0165	0.0004	0.0010	0.0007

Model 1 is a non-adjusted model. Model 2 is adjusted for age. Model 3 is adjusted for model 2 + body mass index, smoking, drinking, hypertension, and diabetes mellitus. Model 4 is adjusted for model 3 + cardiovascular disease.

Table S4. Multivariate regression analyses for association between hyperuricemia and heart rate irregularity in female population.

	Prevalence % (SE)	OR (95% CI)			
		Model 1	Model 2	Model 3	Model 4
Uric acid, continuous (per mg/dL)		1.31 (0.86-2.00)	1.18 (0.81-1.73)	1.21 (0.83-1.78)	1.06 (0.74-1.51)
p		0.2068	0.3927	0.3187	0.7584
Hyperuricemia					
No	0.59 (0.12)	1.00 (ref.)	1.00 (ref.)	1.00 (ref.)	1.00 (ref.)
Yes	2.51 (1.09)	4.33 (1.64-11.43)	3.09 (1.16-8.27)	3.40 (1.18-9.84)	2.50 (0.80-7.77)
p	0.0011	0.0032	0.0243	0.0240	0.1129

Model 1 is a non-adjusted model. Model 2 is adjusted for age. Model 3 is adjusted for model 2 + body mass index, smoking, drinking, hypertension, and diabetes mellitus. Model 4 is adjusted for model 3 + cardiovascular disease.

Table S5. Odds ratio for heart rate irregularity according to uric acid level.

	Prevalence % (SE)	OR (95% CI)			
		Model 1	Model 2	Model 3	Model 4
Uric acid level					
<3 mg/dL	0.50 (0.37)	1.00 (ref.)	1.00 (ref.)	1.00 (ref.)	1.00 (ref.)
3-6 mg/dL	0.76 (0.10)	1.52 (0.35-6.61)	1.43 (0.33-6.24)	1.58 (0.36-6.97)	1.59 (0.36-7.04)
6-9 mg/dL	1.47 (0.25)	2.95 (0.66-13.21)	2.80 (0.60-13.02)	3.27 (0.67-16.05)	3.31 (0.68-16.27)
>9 mg/dL	3.34 (2.01)	6.83 (1.03-45.32)	6.25 (0.92-42.38)	6.24 (0.79-49.51)	7.24 (1.03-51.08)
p for trend	0.0011	0.0005	0.0022	0.0017	0.0019

Model 1 is a non-adjusted model. Model 2 is adjusted for age and sex. Model 3 is adjusted for model 2 + body mass index, smoking, drinking, hypertension, and diabetes mellitus. Model 4 is adjusted for model 3 + cardiovascular disease.
